# Molecular characterization of hepatocarcinogenesis using mouse models

**DOI:** 10.1242/dmm.017624

**Published:** 2015-07-01

**Authors:** Wei Wei Teoh, Min Xie, Aadhitthya Vijayaraghavan, Jadegoud Yaligar, Wei Min Tong, Liang Kee Goh, Kanaga Sabapathy

**Affiliations:** 1 Division of Cellular & Molecular Research, Humphrey Oei Institute of Cancer Research, National Cancer Centre, 11 Hospital Drive, 169610, Singapore; 2 Cancer and Stem Cell Biology Program, Duke-NUS Graduate Medical School, Duke-NUS Graduate Medical School, 8 College Road, 169857, Singapore; 3 Laboratory of Molecular Imaging, Singapore Bioimaging Consortium, 11 Biopolis Way, Helios, 138667, Singapore; 4 Institute of Basic Medical Sciences School of Basic Medicine, Chinese Academy of Medical Sciences, Peking Union Medical College 5, Dong Dan San Tiao, Beijing 100005, China; 5 Department of Biochemistry, National University of Singapore, 8 Medical Drive, 117597, Singapore

**Keywords:** Aflatoxin B_1_, Hepatitis B surface antigen, Hepatocellular carcinoma, Mouse models

## Abstract

Hepatocellular carcinoma (HCC) is a deadly disease, often unnoticed until the late stages, when treatment options become limited. Thus, there is a crucial need to identify biomarkers for early detection of developing HCC, as well as molecular pathways that would be amenable to therapeutic intervention. Although analysis of human HCC tissues and serum components may serve these purposes, inability of early detection also precludes possibilities of identification of biomarkers or pathways that are sequentially perturbed at earlier phases of disease progression. We have therefore explored the option of utilizing mouse models to understand in a systematic and longitudinal manner the molecular pathways that are progressively deregulated by various etiological factors in contributing to HCC formation, and we report the initial findings in characterizing their validity. Hepatitis B surface antigen transgenic mice, which had been exposed to aflatoxin B_1_ at various stages in life, were used as a hepatitis model. Our findings confirm a synergistic effect of both these etiological factors, with a gender bias towards males for HCC predisposition. Time-based aflatoxin B_1_ treatment also demonstrated the requirement of non-quiescent liver for effective transformation. Tumors from these models with various etiologies resemble human HCCs histologically and at the molecular level. Extensive molecular characterization revealed the presence of an 11-gene HCC-expression signature that was able to discern transformed human hepatocytes from primary cells, regardless of etiology, and from other cancer types. Moreover, distinct molecular pathways appear to be deregulated by various etiological agents en route to formation of HCCs, in which common pathways converge, highlighting the existence of etiology-specific as well as common HCC-specific molecular perturbations. This study therefore highlights the utility of these mouse models, which provide a rich resource for the longitudinal analysis of molecular changes and biomarkers associated with HCC that could be exploited further for therapeutic targeting.

## Abstract (precis)

**Summary:** We present initial data from systematic and longitudinal analyses of hepatocarcinogenesis using mouse models that reflect the human disease. This provides a rich resource for the analysis of biomarkers and sequential molecular alterations during disease progression.

## INTRODUCTION

Hepatocellular carcinoma (HCC) is the second-most common global cause of cancer death. It is endemic in Asia (Forner et al., 2012; Tsukuma et al., 2005) and is linked to multiple etiological factors, including diet, alcohol, hepatotoxins, inflammation and microorganisms (El-Serag, 2011). Although molecular advances over the last decade have led to an increased understanding of the genetic changes that occur in HCC, there is still inadequate knowledge about the full spectrum of molecular pathways that are deregulated during the course of hepatocarcinogenesis (Alves et al., 2011; Minguez et al., 2009). This void is reflected in the dearth of molecular pathways that are amenable for effective therapeutic intervention, as well as the lack of biomarkers for early detection of HCC.

The majority of HCC patients in Asia harbor the hepatitis B virus (HBV) (South-east Asia, Taiwan and China) or the hepatitis C virus (HCV) (Japan and Korea) (Kao et al., 2000; Yuen et al., 2009). HBV and HCV carriers are at a higher risk of HCC than non-carriers, highlighting a crucial role for the hepatitis B and C viral components in HCC formation (Kao et al., 2000). Epidemiological studies also suggest a strong relationship between factors such as alcohol consumption, exposure to the fungal aflatoxin B_1_ (AFB_1_), high-fat diet, microbes and other causes of cirrhosis, and HCC formation (Berasain et al., 2009; Fagoonee et al., 2001; Hill-Baskin et al., 2009; Kew, 2003; Wild and Montesano, 2009). However, the detailed knowledge of the effect of these environmental risk factors, either alone or in combination, on the various molecular pathways they subvert in contributing to HCC development is poorly understood, owing to the lack of systematic analysis of their roles in disease progression. Moreover, it is still relatively unclear whether the various etiological factors eventually lead to a convergence of signaling pathways, such that a common molecular HCC signature would emerge and could serve to differentiate HCCs from other cancers to allow their effective targeting.

Many animal models have been generated over the past two decades to model HCC. These include transgenic mice overexpressing factors such as TGFα and c-Myc, or viral factors, such as HCV core protein, Hbx antigen and HB surface antigen (HBsAg) (Calvisi and Thorgeirsson, 2005; Chisari et al., 1985; Ghebranious and Sell, 1998; Moriya et al., 1998; Zhu et al., 2004). Moreover, many chemically induced HCC models have also been utilized to study the progression of the disease (Newell et al., 2008). Some of these models resemble human HCCs and have contributed to varying degrees towards understanding the mechanisms and genes involved in HCC formation, highlighting the relevance of such mouse models. However, many have failed to faithfully recapitulate all pathological changes that occur when a normal hepatocyte undergoes the transformation process to a malignant HCC with all the genetic changes that have been identified in human HCCs. Moreover, the few available, relevant models have not been characterized systematically to realize their relevance to human HCCs, and have therefore not contributed significantly to the identification of biomarkers that may be useful in the early detection of HCCs, or towards pathways that could be therapeutically targeted.

**TRANSLATIONAL IMPACT**

**Clinical issue**

Hepatocellular carcinoma (HCC) is the second-most common leading global cause of cancer-related deaths. It is linked to multiple etiological facts, including infection with hepatitis B virus (HBV) or hepatitis C virus (HCV), a high-fat diet, alcohol consumption and exposure to hepatotoxins such as aflatoxin B_1_ (AFB_1_). HCC is a silent disease that is often only detected late when treatment options are limited. Late detection of HCC is primarily due to the lack of effective prognostic biomarkers. Although several animal models of HCC have been generated over the past two decades, many fail to recapitulate all pathological changes that have been identified in human HCC. Moreover, even the few available relevant models have not been systematically interrogated to understand the progression of the disease better or to identify biomarkers that could be used to detect HCC at an early stage.

**Results**

Here, the authors explore the possibility of utilizing mouse models to perform a systematic and longitudinal analysis of HCC development. Specifically, the authors undertake a longitudinal histological and molecular analysis of hepatocarcinogenesis regulated by HBV alone, AFB_1_ alone or both these etiological factors in combination, by treating transgenic mice expressing hepatitis B surface antigen (an established model for human hepatitis) with AFB_1_. Tumors from animals treated with various etiologies resemble human HCCs histologically and at the molecular level. Moreover, extensive transcriptomic analyses of the models identify an 11-gene HCC expression signature that is able to distinguish HCCs from normal livers, regardless of etiological factors, and from cancers derived from other cell types.

**Implications and future directions**

These initial data demonstrate the similarity and relevance of these mouse models to human HCC, and highlight the usefulness of these models for the longitudinal analysis of the molecular changes associated with HCC. Further in-depth interrogation should increase understanding of the pathways deregulated during HCC development. In addition, exploration of these models should facilitate the identification of both molecular targets for the treatments of HCC and biomarkers that will enable early detection of HCC. Finally, use of these models should allow researchers to analyze systematically the processes that are deregulated by other environmental factors involved in the development of HCC.

In this respect, we have been trying to model human HCC in mice, to systematically study the effects of various environmental factors on both hepatitis-dependent and -independent HCC formation. Importantly, the major eventual goal is to identify biomarkers that could be used to predict the presence of liver malignancies at an early stage. As an initial step, we have started analyzing the effects of AFB_1_, a toxin highly associated with HCC in Asia and Africa (Wu and Santella, 2012), on hepatitis B carriers. We used the HBsAg mice (Chisari et al., 1985), which have been utilized for over two decades and which mimic healthy human hepatitis carriers. Our intention was to perform a longitudinal and systemic investigation to examine the utility of this system to characterize the step-wise events that lead to hepatocarcinogenesis. This report presents our initial molecular analyses of hepatocarcinogenesis regulated by hepatitis alone, AFB_1_ exposure alone or both in combination, which highlights the resemblance of these models to the human HCC context, and therefore promises to provide useful insights in the future.

## RESULTS

### Study design for longitudinal analysis of hepatocarcinogenesis

**Fig. 1.**
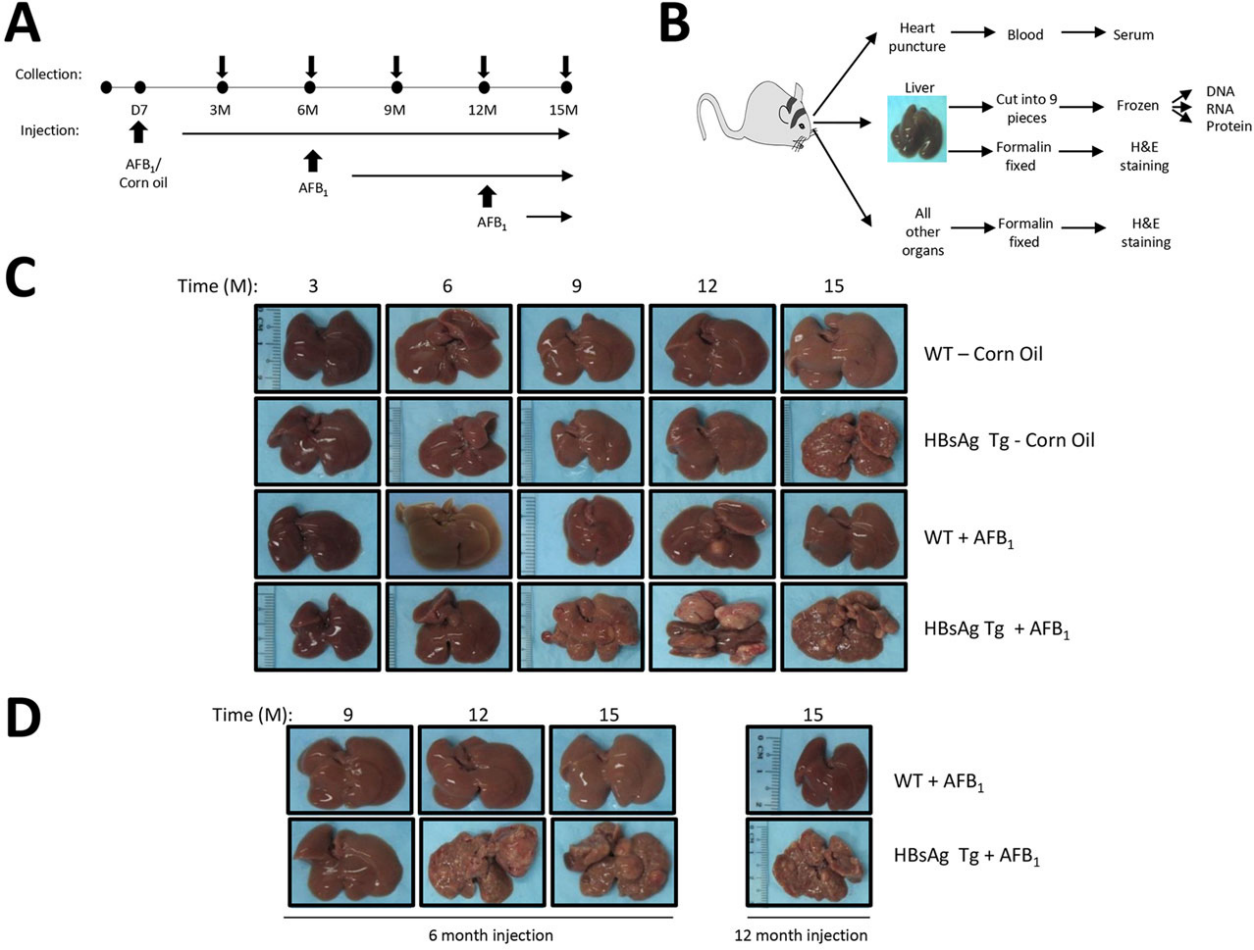
**Experimental set-up and characterization of liver nodules.** (A) Schematic showing the time points of mice collection as well as injections of either corn oil (as control) or AFB_1_, into the various groups of mice. In the first group, 7-day-old (D7) mice were injected and the cohorts were collected at 3-month intervals over 15 months. In the adult group, 6- or 12-month-old (6 M or 12 M) mice were injected and collected at 3-month intervals until 15 months of age. (B) Work flow upon sacrifice of mice at collection. All organs were harvested and fixed for histological analysis by H&E staining. The liver was separated into nine parts, in triplicates, for DNA, RNA and protein isolation, in addition to the histological analysis. Liver nodules were separated from normal livers and collected similarly. Serum was obtained via blood collected through heart puncture upon sacrifice of the animals. (C,D) Liver samples were photographed prior to dissection at each stage of collection [shown as months (M)]. C shows livers from the various mice cohorts that were AFB_1_/corn oil-injected at D7. D shows adult mice when injected at 6 M (left) or 12 M (right). Representative pictures are shown.

We performed a systematic longitudinal analysis of molecular and physiological changes that occur during the process of HCC formation, under conditions that mimic the absence or presence of hepatitis, or upon exposure to the hepatotoxin AFB_1_ at various stages in life. HBsAg transgenic mice and their matched wild-type (WT) counterparts were treated with AFB_1_ on day 7 (D7) after birth, when the liver is at the proliferating stage (Shupe and Sell, 2004), or at adulthood at 6 or 12 months of age (Fig. 1A). Efficacy of AFB_1_ injection to induce DNA adducts was confirmed by southwestern blotting using specific antibodies against the AFB_1_-formamidopyrimidine adducts (supplementary material Fig. S1). Approximately 15-25 mice per group were collected periodically at 3-month intervals, up until 15 months, when all mice were finally sacrificed (Fig. 1A). A total of 727 mice, with about equal numbers of male and female mice, were used to determine the effect of gender on HCC formation (supplementary material Table S1A and B).

Livers and all other organs were collected, together with serum, from all animals at all time points (Fig. 1B). In the event that mice developed liver adenoma or carcinoma, distinct tumor nodules were collected separately from the surrounding liver tissue. Initial histological analysis was performed using livers and all organs from at least six mice per group. Liver tissues were stored frozen in nine separate parts as a reservoir bank for subsequent RNA and genomic DNA analysis.

### AFB_1_ exposure synergizes with hepatitis in inducing cancers only when the liver is proliferating

Time-course analysis indicated that corn oil-treated control HBsAg mice generally developed overt liver lesions beginning from 9-12 months, unlike WT mice, which were without any observable lesions until 15 months (Fig. 1C). By contrast, WT mice injected with AFB_1_ on D7 developed fewer liver lesions. However, HBsAg mice injected with AFB_1_ developed the highest number of liver lesions, macroscopically obvious from around 9 months of age (beginning from 6 months), consistent with epidemiological data (Wild and Montesano, 2009). Analysis of WT mice injected with AFB_1_ at 6 or 12 months did not show any obvious liver lesions until 15 months (Fig. 1D). However, HBsAg mice injected with AFB_1_ at adulthood showed an increase in liver lesions compared with untreated mice (compare Fig. 1C and D), indicating that the quiescent non-proliferating liver (in the case of WT mice) does not provide a permissive environment for liver lesions to be induced by AFB_1_ exposure.

**Fig. 2.**
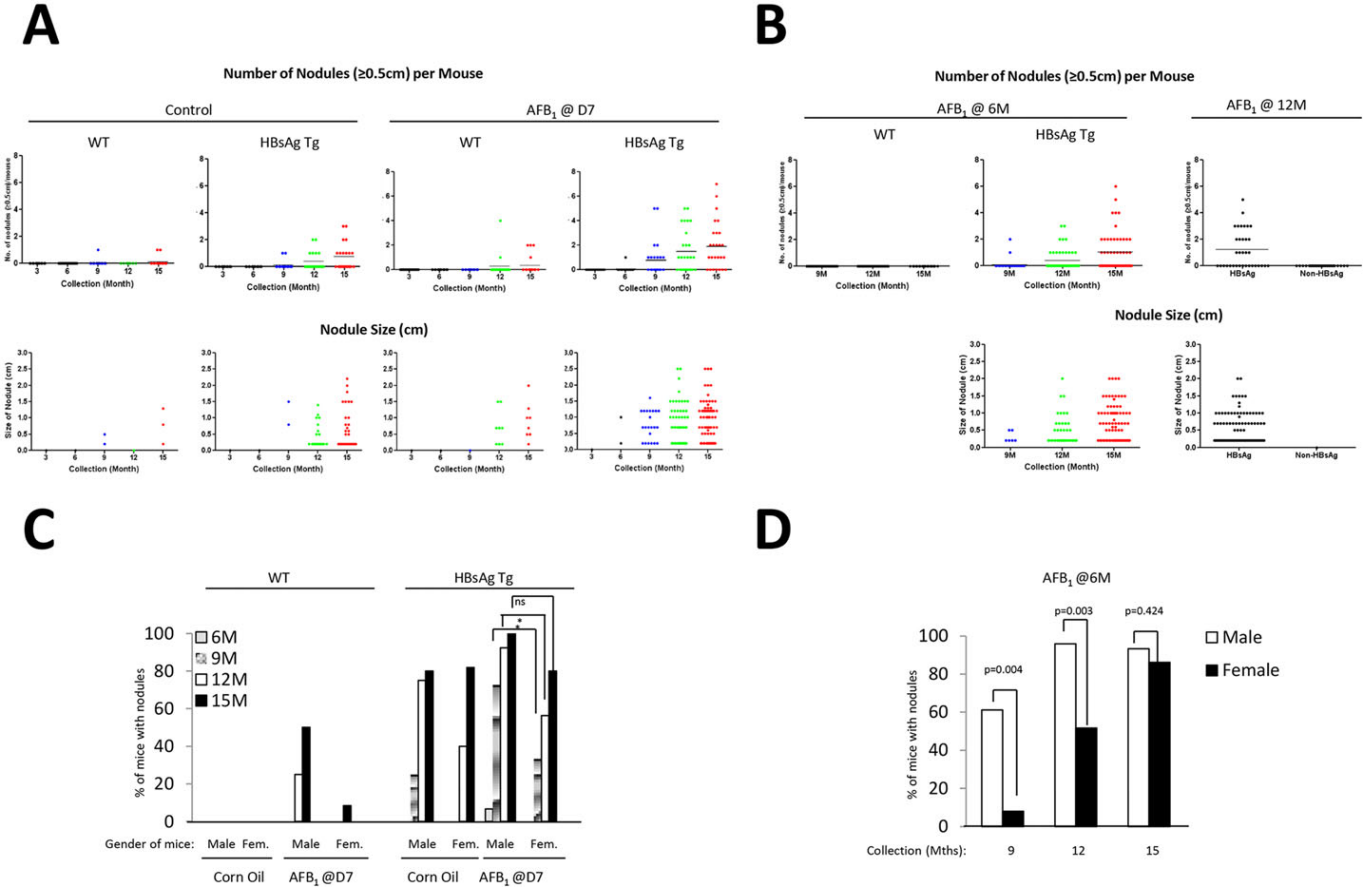
**Characterization of susceptibility based on age and gender.** (A,B) Liver samples were scored as described in the text, and the data are represented as number of liver nodules (≥0.5 cm) per mouse, over the time course of collection in the various groups of mice (top panel). The bottom panel shows the size of these liver nodules in the various categories of mice. Nodules <0.5 cm were scored as 0.2 cm for ease of enumeration, and were counted in this case. Data for mice injected at D7 (A) and 6 M or 12 M (B) are shown. Each dot represents a single mouse (top) or nodule (bottom). Horizontal bars represent average numbers of nodules (top panel). (C,D) Effect of gender on liver nodule development was determined by scoring the percentage of male or female mice with nodules (0.2 cm and above) at the various time points of collections, after injection at D7 (C) or at 6 M (D). *P≤0.05; ns, not significant.

To obtain a detailed understanding on the type of lesions induced, we scored the liver nodules by size. Analysis of large nodules that are ≥0.5 cm indicated that the corn oil-treated control HBsAg transgenic mice themselves developed nodules at 9 months of age, in contrast to WT mice injected with AFB_1_ (Fig. 2A). However, such lesions were visible even at 6 months in HBsAg mice injected with AFB_1_. In general, the average numbers of nodules/mouse were much greater in HBsAg mice injected with AFB_1_, followed by control HBsAg mice and WT mice injected with AFB_1_ [mean nodule numbers (≥0.5 cm)/mouse at 15 months were 2, 1 and 0.2, respectively] (Fig. 2A, top panels). Consistently, the size of nodules mirrored the pattern of nodule abundance in each category. For ease of enumeration, we qualified all small nodules smaller than 0.5 cm as 0.2 cm. Control HBsAg mice developed some large nodules (ranging from 0.2 cm to 2.2 cm, average of 0.6 cm, at 15 months), with most being small (Fig. 2A, bottom panels). AFB_1_ injection alone of WT mice led to fewer nodules that averaged 0.8 cm. By contrast, the bulk of the nodules in HBsAg mice injected with AFB_1_ were larger, ranging from 0.2 to 2.5 cm, with an average size of 1.0 cm (Fig. 2A, bottom panels). Detailed statistical analysis of the number of mice that contained liver nodules confirmed the synergistic effects of HBsAg and AFB_1_ over the individual factors, especially at 9 months after treatment (supplementary material Table S2A). Moreover, the hepatitis component provided by the HBsAg mice was more tumorigenic than the AFB_1_ exposure, as there was a significant difference at 9 months in the HBsAg mice when compared with control mice, whereas it took longer for the AFB_1_-treated WT mice to develop tumors (supplementary material Table S2B).

Abundance and size of liver lesions in adult HBsAg mice exposed to AFB_1_ also increased over time after treatment (Fig. 2B). Importantly, comparison of HBsAg mice treated with AFB_1_ at 6 months and the control (corn oil) group indicated that AFB_1_ treatment indeed accelerated the liver lesions in the HBsAg background of older mice (percentage of mice with lesions ≥0.5 cm at 15 months: untreated versus AFB_1_ at d7 versus AFB_1_ at 6 m: 58.8 versus 73.0 versus 72.0) (Fig. 2B; supplementary material Fig. S2). Although AFB_1_ injection at 12 months did not significantly accelerate the number of nodules present (percentage of mice with lesions ≥0.5 cm at 15 months – untreated versus AFB_1_ at 12 m: 58.5 versus 51.4), the number of mice having ≥2 nodules was higher (4/19, 21% for D7 injections versus 13/35, 37% for 12 M injection) (supplementary material Fig. S2).

Altogether, these data indicate that AFB_1_ exposure requires livers to be at a proliferative state for lesions to form, as adult WT mice without any perturbations were more resistant to liver lesions than HBsAg mice. Moreover, synergistic effect of hepatitis and AFB_1_ was evident regardless of age of exposure.

### Gender bias in formation of liver lesions

Gender bias has been noted in HCC formation in humans, with males being predisposed to the disease (Li et al., 2012). Similarly, liver lesions in our model also showed a gender bias in all cases. Male WT neonatal mice exposed to AFB_1_ had a clear tendency to develop lesions earlier than female mice (Fig. 2C). Similarly, lesions in HBsAg mice developed much earlier in males than in females (*P*=0.0443 at 12 months). However, by 15 months, female mice had an about equal frequency of liver lesions as male mice (*P*=0.226). A similar trend was seen in adult HBsAg mice injected with AFB_1_ at 6 months, with male mice being more susceptible to liver lesion much earlier than female mice; although by 15 months, almost all mice of both genders had similar incidence rates (*P*=0.004 and *P*=0.003 for 9 and 12 months, respectively, and *P*=0.424 at 15 months) (Fig. 2D).

### Histological analysis of mouse and human HCCs

**Fig. 3.**
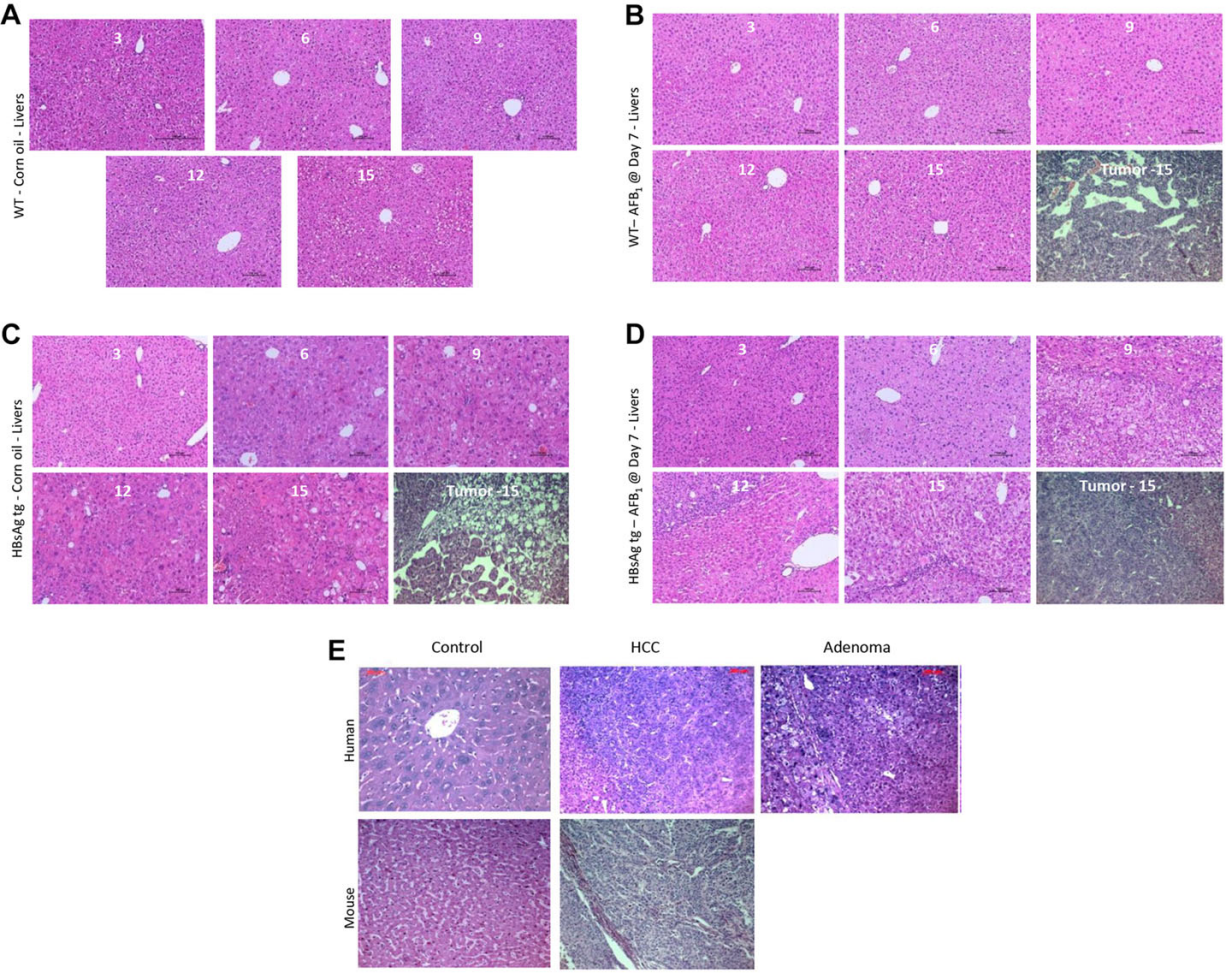
**Histological analysis of liver sections.** (A-D) Liver samples were H&E-stained and analyzed by photomicrography from the various categories of mice injected at D7, at the different time points of collection. Representative pictures from at least six independent mice for each time point/category are shown. Liver tumors from each case, at 15 M of age, are shown in the last image of B-D (denoted by ‘Tumor - 15’). (E) Comparison of mouse liver nodules at 15 M with human HCC and adenoma.

Histological analysis was performed using 6-14 mice per group to determine the status of the liver lesions classified as regenerative foci (F), adenoma (A) or carcinoma (H). Control WT mice showed signs of degenerative changes only at 15 months of age (Fig. 3A). However, livers from control HBsAg mice were composed of large hepatocytes which varied in size and shape, with nuclear pleomorphism and vacuolated cell foci (Fig. 3B). AFB_1_ injection did not alter the morphology of the normal livers of mice significantly as compared with the effects of HBsAg (compare Fig. 3A with C, and Fig. 3B with D). HCCs arising in the various groups of mice at 15 months indicated that they were histologically similar. Comparison of human HCC with mice HCCs from this study also indicated that they were strikingly similar (Fig. 3E).

Based on the histological analysis, control HBsAg mice generally developed only foci or adenomas (2 and 4 out of 12, respectively, at 15 months of age) (Table 1A), as has been previously described (Ghebranious and Sell, 1998). AFB_1_ exposure at D7 generally led to foci and carcinoma formation in WT mice (5 and 1 out of 10, respectively, at 15 months of age). By contrast, HBsAg mice injected with AFB_1_ had more cases of carcinoma, together with adenoma and foci of alteration (3 out of 11 mice each), indicating that the severity of liver lesions correlates with the synergistic effects of hepatitis and AFB_1_, concurring with the gross morphological analysis (Fig. 1C,D). Similarly, adult HBsAg mice injected with AFB_1_ also developed carcinomas in addition to adenomas and foci, much more frequently compared with the group without AFB_1_ injection (Table 1B). By contrast, WT mice did not develop any lesions, again highlighting the ability of AFB_1_ to induce lesions only on an HBsAg background at adulthood.

**Table 1.**
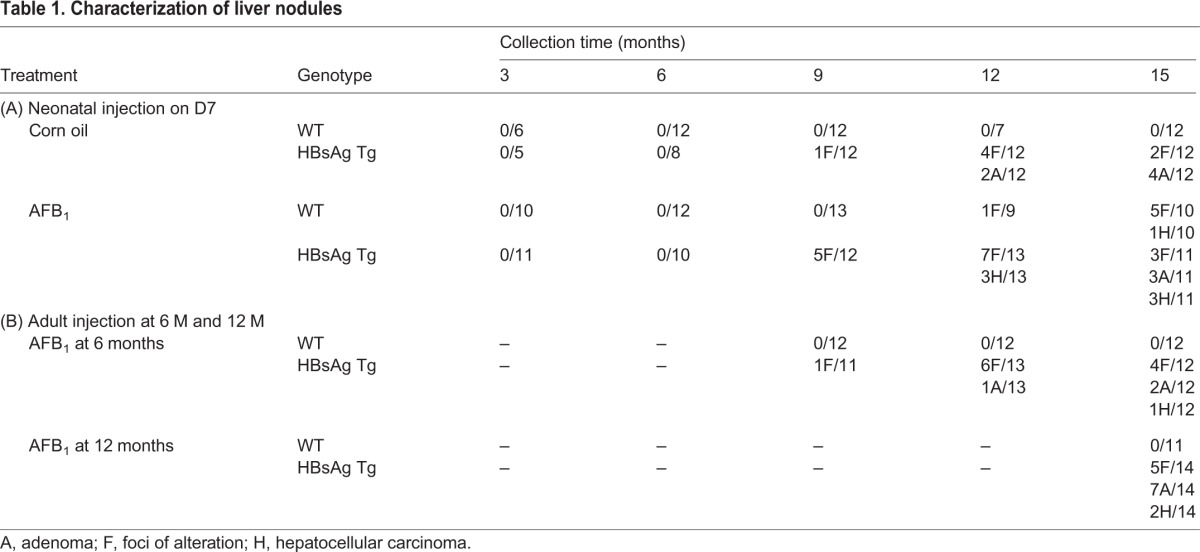
**Characterization of liver nodules**

### Transcriptomic analysis identifies HCC-specific molecular signature independent of etiological factors

Given that the tumors that arise in the WT or HBsAg mice treated with AFB_1_ appear histologically similar, we next evaluated whether the molecular profiles of tumors or normal tissues from the various etiologies would also be similar. To this end, we performed an mRNA profiling experiment using samples from the following categories of 15-month-old mice: histologically normal liver tissues from control or AFB_1_-treated WT mice and control HBsAg mice; normal tissue adjacent to the tumor nodules from AFB_1_-treated HBsAg mice; and, finally, tumor nodules from control HBsAg mice, or from AFB_1_-treated WT or HBsAg mice [total of seven categories: WT_Oil_N (*n*=3), WT_AFB_N (*n*=2), HBsAg_Oil_N (*n*=3), WT_AFB_T (*n*=3), HBsAg_Oil_T (*n*=2), HBsAg_AFB_AN (*n*=3) and HBsAg_AFB_T (*n*=4)].

**Fig. 4.**
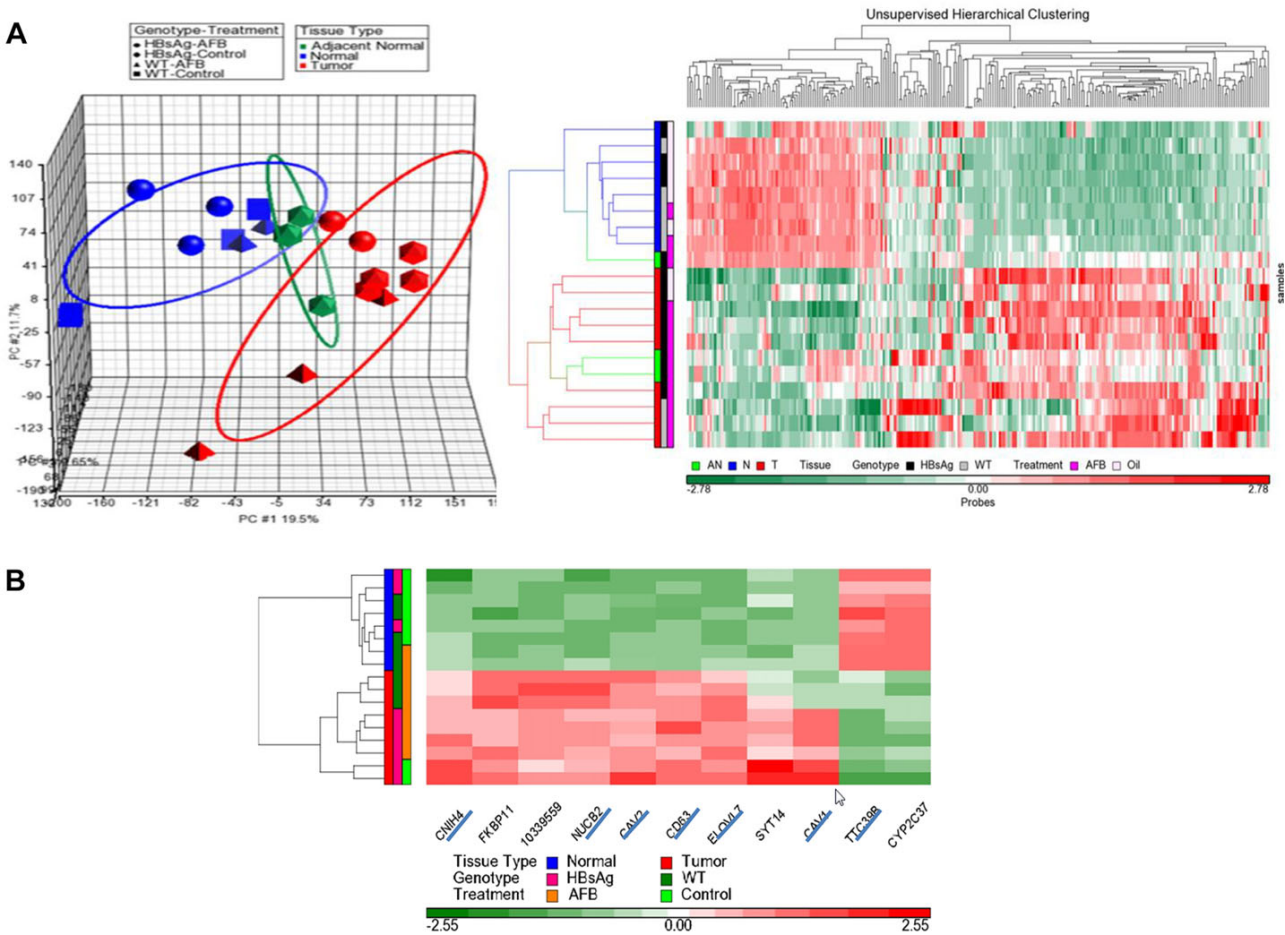
**Genomic characteristics of mouse HCC model.** (A) Transcriptome profiling of normal livers and liver nodules from the various groups were performed by Affymetrix mouse arrays. PCA, shown in the left panel, revealed distinct clustering of tumor and normal samples, with adjacent normal clustered between tumor and normal samples. Each data point represents one sample and the ellipsoids represent the zone within 2 s.d. of each experimental group. Right panel shows the heat map of unsupervised hierarchical clustering based on probes with s.d.≥1, which reveals distinct clustering of tumor and normal samples. Within the tumor group, differential clustering was also observed based on genotype. Treatment parameters are indicated on the sides. (B) Heat map of 11 genes found to be differentially expressed between HBsAg_Oil_T and HBsAg_Oil_N in the mouse HCC model. Distinct clustering was observed as expected between tumor and normal samples, based on the expression pattern of these genes. Seven genes (underlined) could be mapped to human microarray chips.

A principal component analysis (PCA) indicated that normal livers from the three groups (etiologies) clustered together (*n*=8), distinct from the tumor group (*n*=9) (Fig. 4A, left panel). Normal tissue adjacent to the tumor nodules from AFB_1_-treated HBsAg mice formed a distinct intermediate category, indicating a progressive nature of the changes from normal liver to nodules. Unsupervised hierarchical clustering based on 256 probes (s.d.≥1) showed that tumors and normal livers were clustered separately, with subtle differences within each group (Fig. 4A, right panel). Closer inspection of the histologically normal liver cluster indicated subtle clustering based on genotype, attributable to the presence of the HBsAg transgene. Similarly, tumor samples from all three categories also clustered together, being broadly similar. However, closer inspection revealed that the tumor samples were more distinctly clustered, based not only on genotype but also on AFB_1_ exposure (supplementary material Fig. S3). The AFB_1_ tumor signature appeared to be distinct from the HBsAg tumor signature, and tumors from HBsAg mice treated with AFB_1_ had a mix of both signatures (supplementary material Fig. S3), suggesting that treatment of AFB_1_ can accentuate a distinct pathogenic process in tumors.

Differential analyses between HBsAg_Oil_T and HBsAg_Oil_N revealed 11 genes that passed Bonferroni correction, constituting an HCC signature, being able to distinctly segregate the tumors from normal tissues regardless of etiology and inducing factors (Fig. 4B). Two genes, TTC39B (*Ttc39b* – Mouse Genome Informatics Database) and CYP2C37 (*Cyp2c37*), were downregulated in tumors, whereas nine genes (CNIH4/*Cnih4*, CAV2/*Cav2*, CDC63, ELOVL7/*Elovl7*, NUCB2/*Nucb2*, SYT14/*Syt14*, FKBP11/*Fkbp11*, 10339559 and CAV1/*Cav1*) were upregulated. Seven of the genes (all except one were overexpressed in the mouse model) were mappable to human microarray transcripts.

**Fig. 5.**
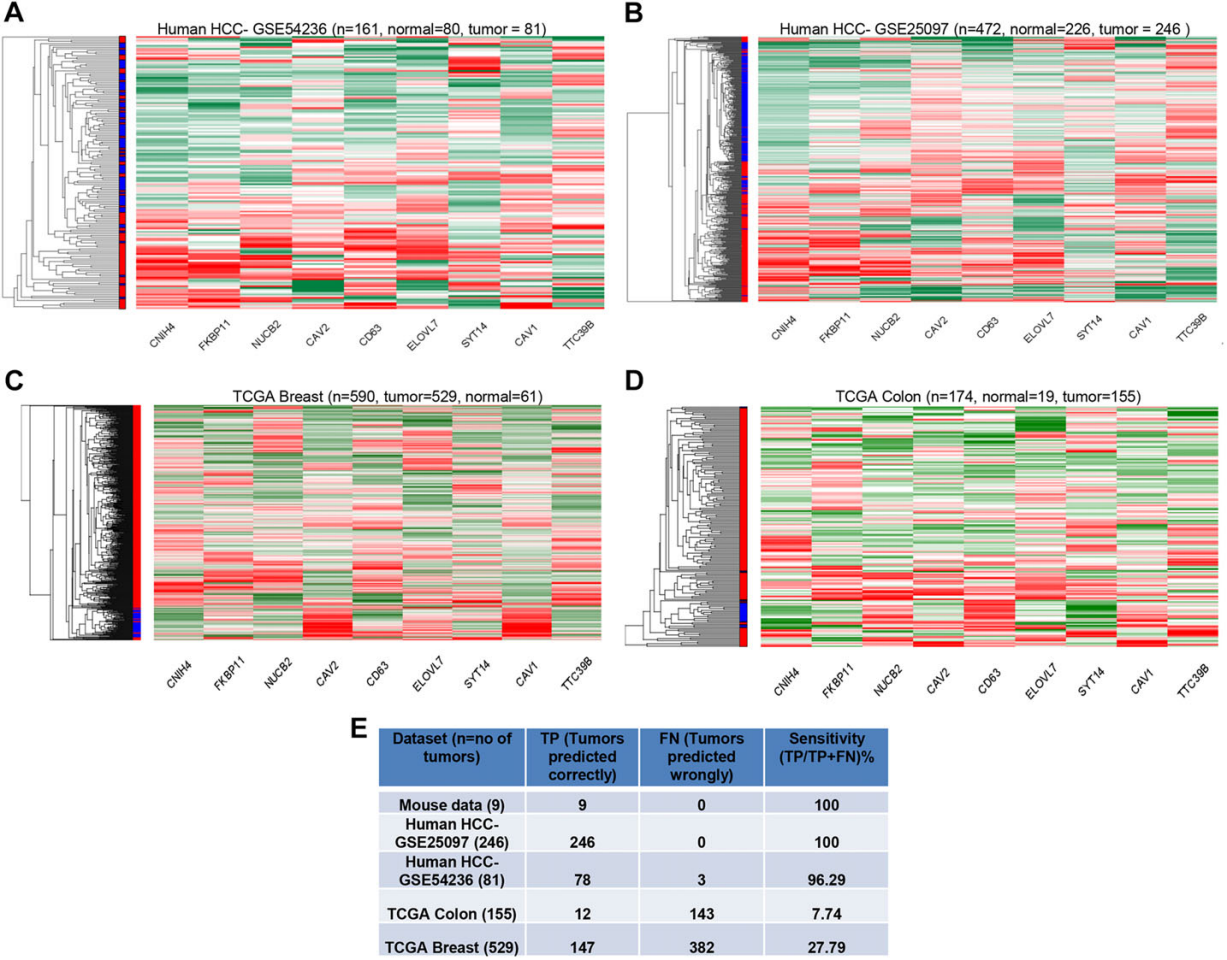
**HCC-specific gene expression signature.** (A,B) Differential clustering between tumor and normal samples was observed when segregated by the HCC gene expression signature, similar to the mouse HCC model. (C,D) Heat map of unsupervised clustering based on the gene signature in human breast cancers (C) or colon cancers (D) showed less differential clustering of tumor and normal samples. (E) Summary of SVM model analysis of sensitivity of the gene expression signature to identify the human tumor types.

We therefore examined whether the HCC gene signature identified in the mouse models here is capable of segregating human HCCs of various etiologies. To this end, we examined three published HCC datasets: one from a European population (that lacks AFB_1_ exposure and HBV; GEO dataset 54236); one from the hepatitis-based Chinese population (GSE25097), and one from the Singaporean Chinese hepatitis-based population lacking AFB_1_ (E-MEXP-84). Interestingly, the gene expression signature was able to distinctly segregate the HCCs from normal tissues in all three cases (Fig. 5A,B; supplementary material Fig. S4A), indicating the robustness of the signature that was not limited by the etiological factors. Importantly, this expression signature was unable to clearly segregate other human cancers such as breast (TCGA dataset) (Fig. 5C) or colon adenocarcinoma (TCGA dataset) (Fig. 5D). To further assess the specificity of this mouse-derived gene expression signature for human HCCs, a support vector machines (SVM) model was generated using the mouse HCC data and then tested on the other human cancer datasets. As shown in Fig. 5E, the mouse HCC model could predict well for human HCCs but not for other cancers, such as breast and colon. For instance, the mouse gene expression signature was able to discern out all 246 HCC tumors from one dataset and 78 of 81 HCCs from another, resulting in over 96% sensitivity. By contrast, the signature only predicted 12 out of 155 colon cancers and 147 out of 582 breast cancer samples, indicative of ∼7.7-27.8% specificity, markedly lower than the values obtained with the HCC datasets.

We also examined the ability of this gene expression signature to distinguish between transformed and untransformed hepatocytes, by analyzing the datasets from several hepatic cell types. Interestingly, our HCC signature was indeed able to segregate out the HepG2 transformed cells from the primary HepRG cells or the embryonic stem cell-derived primary hepatocytes, regardless of AFB_1_ treatment (GSE40117) (supplementary material Fig. S4B). Altogether, the transcriptomic data analyses demonstrate the identification of a gene expression signature from the mouse models that is able to distinguish HCCs from untransformed primary hepatocytes, regardless of the etiological factors, supporting the specificity and relevance of the mouse model to human HCC.

### Distinct pathways lead to hepatocarcinogenesis based on etiological factors

**Fig. 6.**
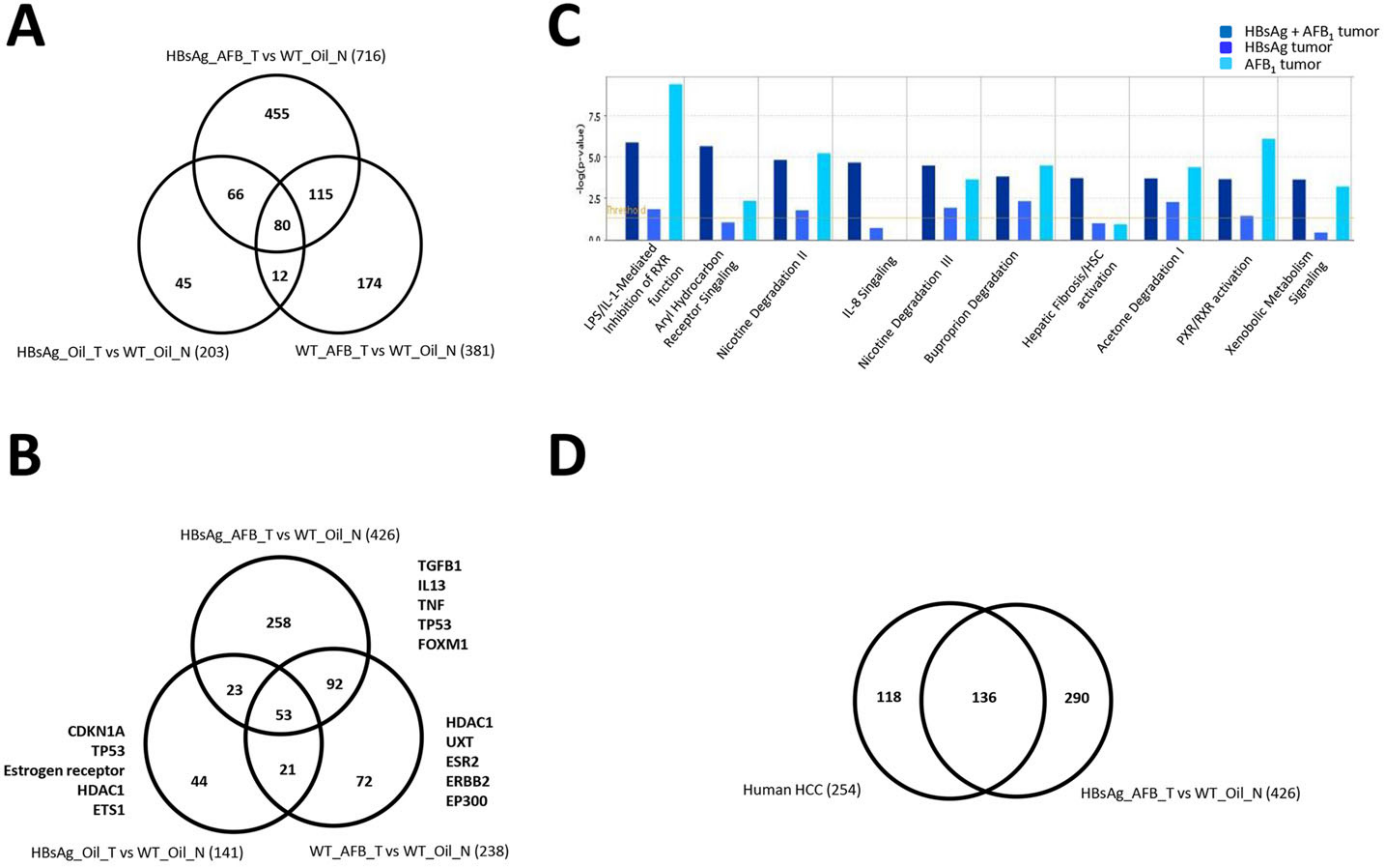
**Molecular characterization of liver nodules from mouse models.** (A,B) Venn diagram showing the genes (A) or upstream regulators (B) affected in the liver tumors in each of the categories and their overlap. The top five upstream regulators are highlighted on the sides. Numbers in parentheses indicate the genes/regulators identified through the differential gene expression. (C) Top canonical pathways involving the upstream regulators in the tumors from the various categories are listed. (D) Venn diagram showing upstream regulators perturbed in human HCCs and HBsAg_AFB_T versus WT_Oil_N.

Although we have noted the presence of a common gene signature that is able to distinguish HCCs regardless of etiological factors, suggesting a convergence of signaling cascades in HCC formation, we also observed that AFB_1_ exposure or the presence of HBsAg has some molecularly distinguishing features (supplementary material Fig. S3). This highlights that the pathways perturbed by these etiological factors are different, alluding to the existence of varying pathogenic processes that lead to HCC formation. We therefore investigated this phenomenon further by analyzing the differences in gene expression from tumors from the three groups (AFB_1_ or HBsAg alone or in combination). Tumors arising in the presence of HBsAg alone led to the lowest number of differentially expressed genes (compared with normal livers), as opposed to those induced by AFB_1_ (203 versus 381 genes) (Fig. 6A). Unsurprisingly, presence of both etiological factors led to the highest number of changes (716 genes altered). Furthermore, the tumors arising due to AFB_1_ or HBsAg exposure alone were more dissimilar from each other than from their common counterparts, further alluding to different pathways perturbed during the carcinogenic process. Nonetheless, 80 genes were commonly perturbed in all tumors, which contained some of the genes that constituted the HCC gene expression signature.

Using these differentially expressed genes, we tested for biological pathways that were affected. As expected, the profiles from the three different groups revealed that upstream regulators of the differentially expressed genes identified by the pathway analysis program (Fig. 6B), and biological pathways they affect (Fig. 6C) could be distinguished in each group. Top upstream regulators deregulated included the cytokines TgfB1, IL-13, TNF, Tp53 and FoxM1, which were regulated mainly by the combined presence of HBsAg and AFB_1_; CDKN1A, estrogen receptor, HDAC1 and ETS1, which were deregulated by the presence of HBsAg alone; and UXT (regulating androgen receptor), ESR2 (estrogen receptor 2 signaling), ERBB1 and P300, which were affected by AFB_1_ alone. An interesting observation was that the tumors arising from HBsAg+AFB_1_ also had different signaling pathways activated compared with individual risk factors, suggesting that the molecular physiology of the tumors is different. Moreover, gross analysis of top canonical pathways affected in these three groups of tumors indicated that there were common as well as distinct pathways affected, based on the etiological factors. There were more commonalities between tumors arising from both HBsAg/AFB_1_ or from AFB_1_ alone, whereas the HBsAg-derived tumors were very distinct (Fig. 6C; supplementary material Fig. S5). Future in-depth analyses of larger sample size will provide further details based on the etiological factors.

Finally, to evaluate the overlap between the biological pathways and processes affected in mouse tumors with human HCCs samples, we compared the transcriptome profiles of human hepatitis-based HCC samples (Wang et al., 2007) with the HBsAg_AFB_T versus WT_Oil_N, which revealed over 50% common upstream regulator pathways (Fig. 6D), highlighting the commonality in pathogenesis of the liver lesions. Consistently, most of the top canonical pathways, such as LPS/IL-1-mediated inhibition of RXR function, nicotine, acetone and buproporin degradation pathways, and PXR/RXR activation, were similar in both mouse and human tumors (supplementary material Fig. S6). Similarly, gene ontology analysis indicated that the biological processes (metabolic and cellular processes), molecular functions (electron carrier activity, binding and catalytic activity) and cellular components (extracellular region and cell junctions) affected by tumor development were similar in all mouse tumors and the human HCCs (supplementary material Fig. S7). This corroborated further that liver tumors from these mouse models are indeed molecularly similar and resemble the human HCCs. Taken together, these data collectively indicate that whereas the biological processes affected due to the various etiological agents en route to hepatocarcinogenesis do differ, highlighting specificity of the inducing agent, there is also a significant degree of overlap, indicative of culmination in a common mode for the transformation of hepatocytes.

## DISCUSSION

The main goal of this preliminary work was to ascertain the similarity of mouse models of hepatocarcinogenesis induced by various etiological factors to human HCCs, such that the models could be eventually used for in-depth interrogation of pathways deregulated during the carcinogenic process as well as for biomarker discovery. As presented, we have used the model that reflects the hepatitis-B carriers, as well as the common hepatotoxin, AFB_1_, both of which are predominant in the Asian and the African context. Histological and molecular transcriptome analyses of end-point tumors indicated that mouse tumors of various etiologies are indeed similar to the human cases. This underscores the significance of these models as suitable tools to understand the molecular mechanisms perturbed under specified conditions in the course of hepatocarcinogenesis. Thus, although these models are not entirely new, they offer a unique opportunity to interrogate in-depth, in a longitudinal manner, the alterations that occur leading to the transformation of the hepatocyte, as well as to identify non-invasive markers that would be apparent during the course of carcinogenesis.

Overtly, two aspects of human HCCs have also been noted in these models. First, the synergistic effect of AFB_1_ with hepatitis, especially in the adult mice, confirms that the underlying alterations in the hepatocytes are essential for manifestation of the tumor-promoting effects of AFB_1_. Consistently, only injections at the neonatal stage, when the liver is proliferating (Shupe and Sell, 2004), had an effect on the non-hepatitis WT mice, which were resistant to AFB_1_ injections at adulthood. Second, the gender bias observed in human HCCs is also recapitulated in these models, emphasizing the notion that HCC formation in a hepatitis background or upon exposure to AFB_1_ works through similar mechanisms and is dictated by gender-specific factors. However, one feature that has emerged is that whereas gender biasness is observed in the course of HCC development, the female mice eventually succumb to a similar extent as males, suggesting that on an age-adjusted basis, older females are as susceptible as males to HCC.

Histological analysis also confirmed that the end-stage tumors induced by the various factors in mice do indeed resemble the human HCCs. Molecular analyses have provided further significant insights into the pathogenesis of the tumors. First, the data presented here have revealed that whereas the end-stage tumors appear to be molecularly similar, the pathways that are induced to result in these tumors could be different and reflect the etiological factors. For example, the effect of AFB_1_ was distinguishable from HBsAg-induced tumors, indicating that the processes deregulated by the viral component are indeed different to that induced by the hepatotoxin. Interestingly, however, combination of these two factors resulted in further distinct pathways that were deregulated, highlighting the possibility to distinguish molecularly the etiological factors responsible for carcinogenesis. Hence, etiological factor-specific molecular stratification could be utilized for identifying causal factors for HCC, which could also be beneficial for designing targeted therapy. Consistently, overall pathway analysis revealed that the pathways affected in the tumors include the inflammatory pathways, estrogen signaling, the p53 pathway and histone modifications, reflecting a systemic nature of the disease that arises from the initial perturbations through the hepatitis component, as well as the hepatotoxins. Thus, these models offer the opportunity for detailed analysis of the genetic and epigenetic alterations that occur sequentially and/or concurrently during the course of HCC development, through the evaluation of earlier time-point biopsies. This would allow us not only to understand the mechanisms involved, but would also provide potential pathways for therapeutic targeting to reduce or inhibit the transformation process.

Second, despite the differences based on etiological factors, the extent of similarities of genes and pathways that are affected indicates a general convergence of pathways leading to hepatocarcinogenesis. The common 11-gene expression signature derived collectively from our mouse models was able to distinguish human HCCs of various etiologies, but was not specific for other cancer types. This signature was also able to distinguish untransformed hepatocytes from transformed cells regardless of AFB_1_ treatment. This indicates that the genetic programs deregulated specifically during hepatocyte transformation can indeed be distinguishable from the general transformation signatures. Importantly, this also suggests that, based on these common pathways, general targeting strategies could be identified to selectively inhibit the ‘transformed’ hepatocyte.

The data also imply that the mouse models described here would be very useful in several aspects. First, given the dearth of biomarkers available to detect HCC during its initial progression, the longitudinal analysis should allow us to identify useful biomarkers that can be tested in the human contexts; second, the HCC-specific gene expression signature could be used in identifying circulating tumor cells from peripheral blood, thereby allowing the early detection of HCCs; and, finally, the collection of the enormous resource from these cohorts would also allow us to interrogate in a systematic manner the processes sequentially deregulated by the various etiological factors that lead to HCC formation.

Altogether, the data presented here demonstrate the similarity and relevance of the mouse models to the human context. A detailed systematic analysis might thus also lead to the discovery of various molecular mechanisms subverted by various other environmental agents, such as high-fat diet, alcohol and others, leading to hepatocarcinogenesis.

## MATERIALS AND METHODS

### Mice and treatment

HBsAg transgenic mice, C57BL/6J-Tg(Alb1HBV)44Bri/J (Jackson Laboratory), were crossed to C57BL/6J wild-type (WT) mice to obtain HBsAg and WT littermate control mice, which were given a single intraperitoneal injection of AFB_1_ [6 µg/g body weight (19 nmol/g) in trioctanoin containing 6% DMSO or corn oil] on day 7 (D7) after birth, as described (Tong et al., 2006), or at 6 or 12 months (M) of age.

Liver samples (nodules and un-inflamed normal areas), all other major organs and serum (by cardiac puncture) were collected at 3-month intervals until 15 months, for histological analysis, RNA extraction, genomic DNA and serum extraction, and were kept as frozen samples or fixed in 4% formalin. All sacrifice was carried out between 11 am and 1 pm for all collections, livers were photographed after sacrifice, and nodules ≥0.2 cm in diameter were counted. All animal experiments were approved by and performed in accordance with the guidelines of the SingHealth Animal Care and Use Committee. Histological analysis is described in the supplementary material.

### Microarray analysis

Between two and four samples per group were used for transcriptome analysis, using the Affymetrix GeneChip Mouse 1.0 ST arrays, as described (Lee et al., 2012). A nomenclature of genotype/treatment/tissue was used to denote the 7 groups: WT_Oil_N (*n*=3), WT_AFB_N (*n*=2), HBsAg_Oil_N (*n*=3), WT_AFB_T (*n*=3), HBsAg_Oil_T (*n*=2), HBsAg_AFB_AN (*n*=3), HBsAg_AFB_T (*n*=4), in which N, AN and T refer to normal, adjacent normal, or tumor tissue, respectively.

PCA and heat map analyses: microarray data were processed with default preprocessing settings using RMA and quantile normalization. Principal component analysis (PCA) was performed using all 35,556 probes. Unsupervised hierarchical clustering was generated using probes with standard deviation (s.d.)≥1. Differential analyses were performed between two groups using ANOVA, and probes were selected based on *P*<1e^−06^ (Bonferroni correction). All analyses were performed on the Partek Genomic Suite (Partek).

Pathway analyses: Ingenuity Pathway Analysis Software (www.ingenuity.com) was used to determine canonical pathways and upstream regulators enriched. Differentially expressed genes in each group were selected using a fold change>2 and FDR<0.05. Upstream regulators of these genes were subsequently elucidated from IPA and compared. Pathways analyses were performed on the upstream regulators.

Human genomics data: Human HCC transcriptome profiles were obtained from three sets of HCCs and matched with normal sets as indicated. Human breast and colorectal datasets were from TCGA. Probes between mouse and human transcripts were mapped based on Refseq gene symbol, resulting in 13,359 common transcripts. Differentially expressed genes were elucidated using ANOVA between tumor and normal samples in each genomics dataset. Our mouse microarray data have been deposited at NCBI/GEO, with the GEOSET number GSE54054.

For analysis of specificity, we used the support vector machine (SVM) (www.chibi.ubc.ca/gist) as modelling method with default settings.

### Statistical analysis

Fisher's exact test was used to analyze the difference in incidences of liver nodules in different experimental groups. Chi-square test was used to analyze the biggest nodule size of each mouse in different experimental groups.

### Histology

Histological analysis was performed on 3-µm-thick sections stained with hematoxylin and eosin (H&E), as described (Tong et al., 2006). Foci of cell alteration have no obvious disruption of the liver architecture, and the affected hepatocytes merge with adjacent hepatocytes without compressing adjacent normal parenchyma. Hepatocellular adenomas are small, formed from closely packed cells, generally larger than the surrounding liver cells without great variability in size. HCC is represented either as a trabecular or solid form compressing adjacent normal parenchyma, with pleomorphic nuclei and aberrant mitotic figures. Human HCC slides were obtained from Peking Union Medical College (Beijing, China).

## Supplementary Material

Supplementary Material
