# Correction: Molecular characterization of hepatocarcinogenesis using mouse models

**DOI:** 10.1242/dmm.050441

**Published:** 2023-10-19

**Authors:** Wei Wei Teoh, Min Xie, Aadhitthya Vijayaraghavan, Jadegoud Yaligar, Wei Min Tong, Liang Kee Goh, Kanaga Sabapathy

An error was noted in *Dis. Model. Mech.* (2015) **8**, dmm017624 (doi:10.1242/dmm.017624).

The authors inadvertently transposed the mouse and human hepatocellular carcinoma (HCC) panels in Fig. 3E during figure assembly. The corrected figure panel is shown below.

**Fig. 3E (corrected panel).**
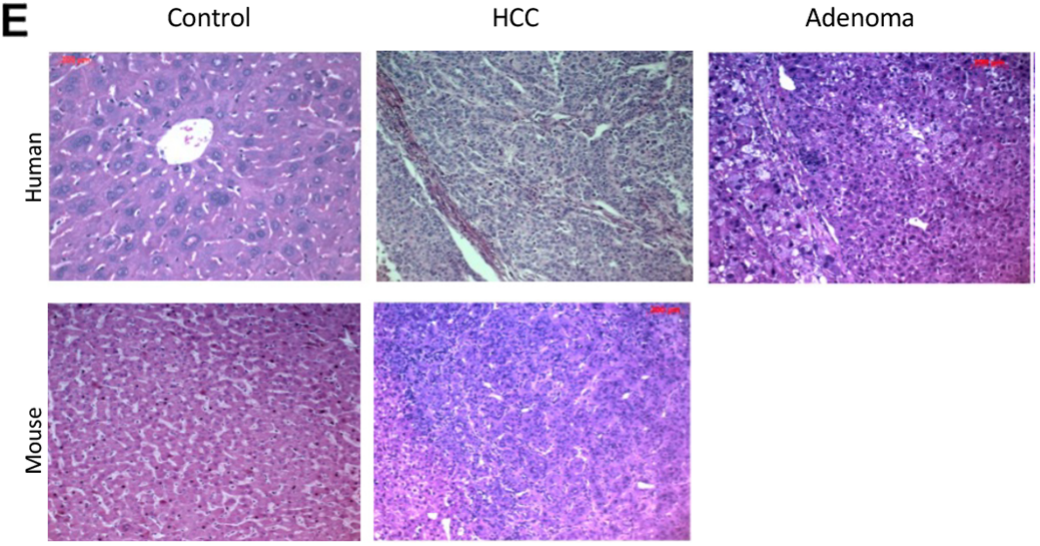
**Histological analysis of liver sections.** (E) Comparison of mouse liver nodules at 15 M with human HCC and adenoma. The mouse HCC image was acquired from the same tumor sample shown in D (‘Tumor – 15’).

Furthermore, the bottom-right image in Fig. 3D (‘Tumor – 15’) and the mouse HCC image in Fig. 3E were acquired from the same tumor sample under different imaging conditions. The legend for Fig. 3E has been updated to reflect this.

This Correction does not affect the results in the article or the conclusions of this study. The authors apologise for the error and any inconvenience it may have caused.

