# Supplementary material for: Molecular characterization of hepatocarcinogenesis using mouse models
Source: Dis Model Mech. 2015 Jul 1;8(7):743–53. doi: 10.1242/dmm.017624 (PMC4486853; doi:10.1242/dmm.017624)
Supplement: Supplementary Material [file supp_8_7_743__index.html]

Supplementary Material 

# Molecular characterization of hepatocarcinogenesis using mouse models

## DMM017624 Supplementary Material

- Supplementary Material
