# Supplementary material for: Molecular characterization of hepatocarcinogenesis using mouse models
Source: Dis Model Mech. 2015 Jul 1;8(7):743–53. doi: 10.1242/dmm.017624 (PMC4486853; doi:10.1242/dmm.017624)
Supplement: Supplementary Material [file supp_017624_DMM017624supp.pdf]

Supplementary figures

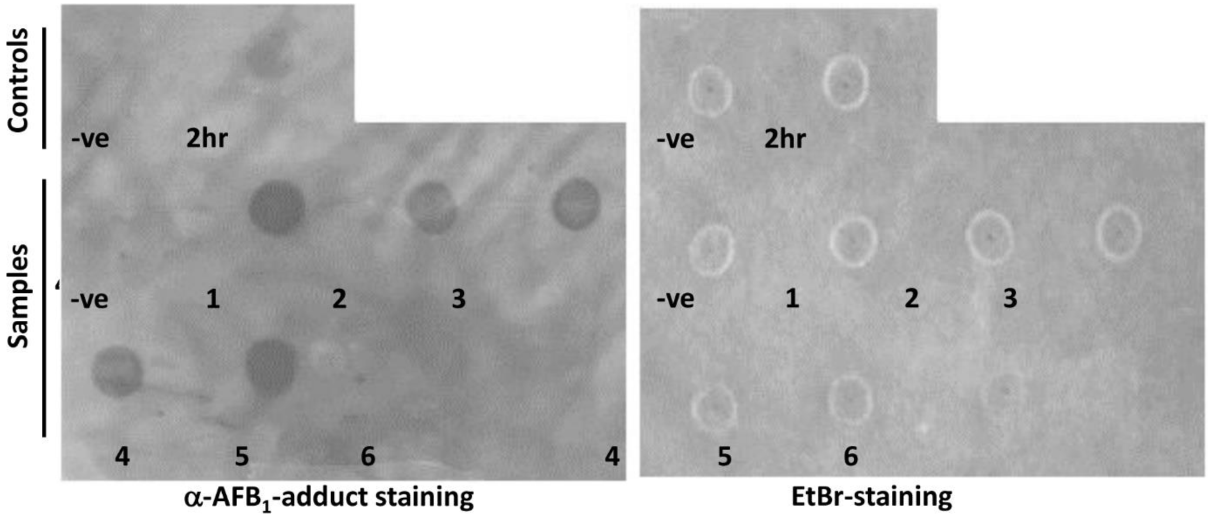

Figure S1. Dot-blot analysis of AFB_1-induced adducts

South-western dot-blot analysis was performed using 4 μg genomic DNA (gDNA) from livers of the control or AFB_1–injected (samples) mice (@D7) and the anti-AFB_1-adduct antibody. Loading for DNA was visualized by ethidium bromide (EtBr) staining. –ve represents without the anti-AFB_1 antibody. gDNA samples 1-6 are from six individual mice, harvested 2 h post AFB_1-injection.

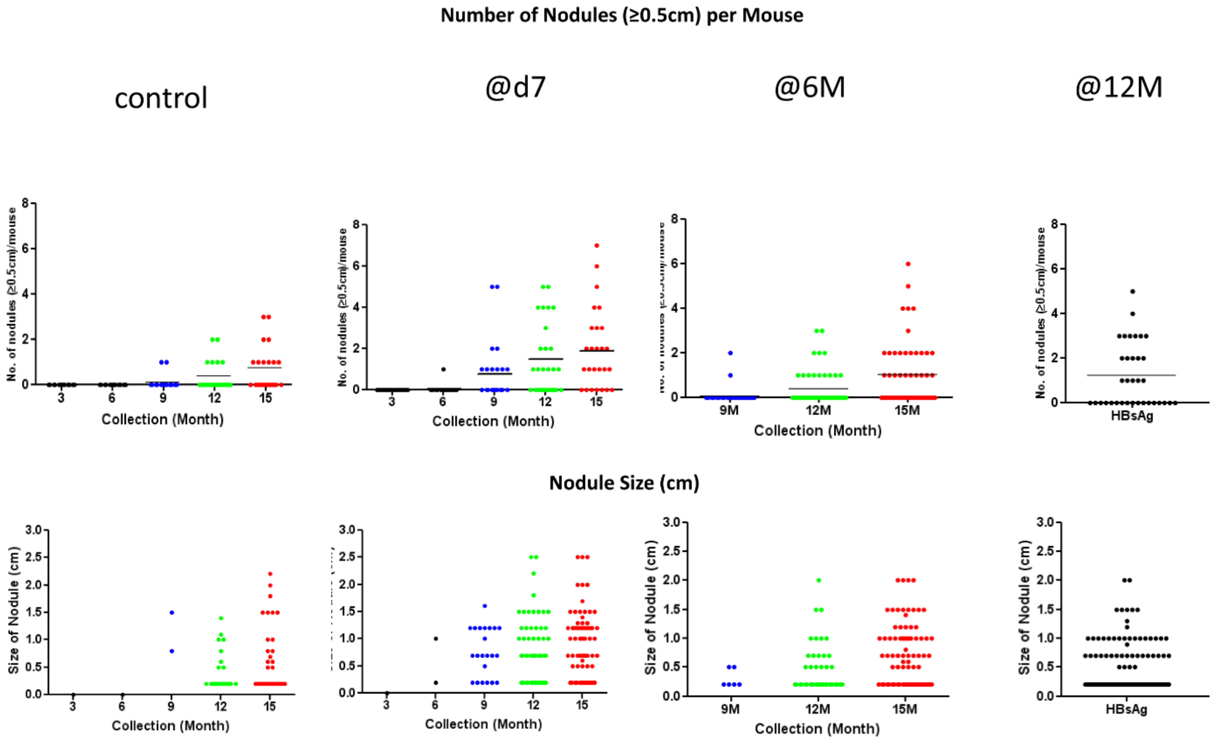

Figure S2. Characterization of liver nodules

Liver samples were scored and plotted as described in the main legends (Figure 2). Direct comparison of HBsAg mice that were AFB$_1$-injected at D7, 6M or 12M are shown, together with D7 oil controls.

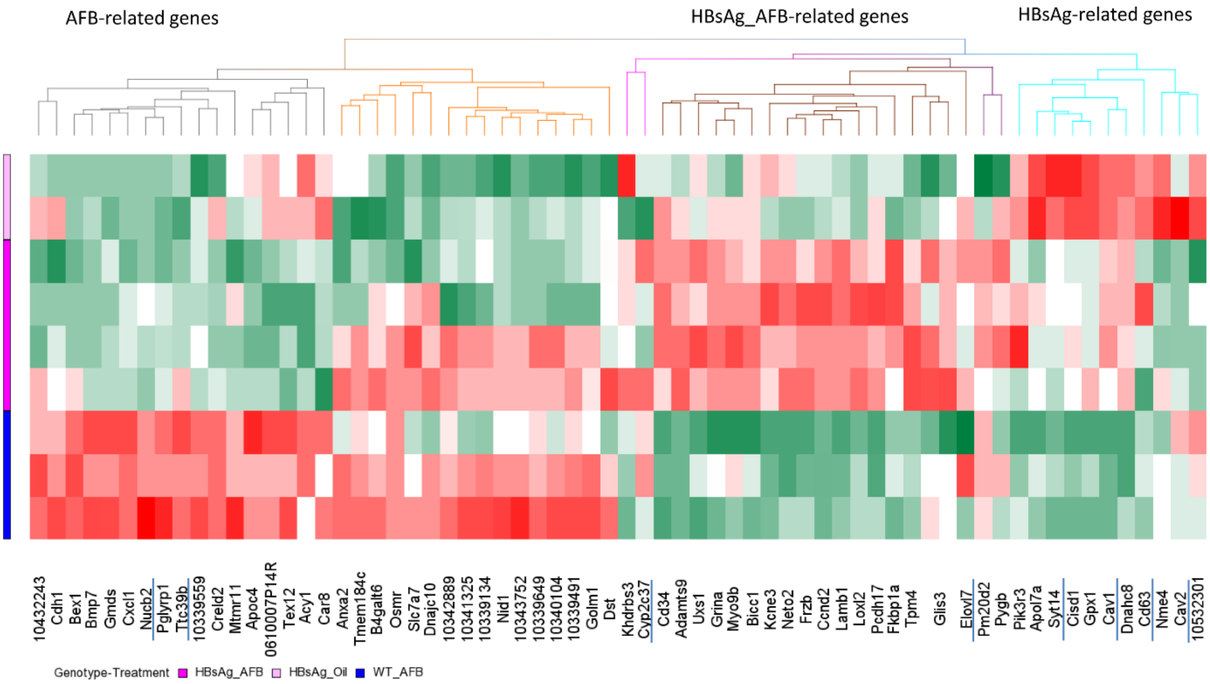

Figure S3. Divergent pathways are deregulated by HBsAg and AFB_1

Genes obtained from HBsAg_AFB_T vs WT_Oil_N, HBsAg_Oil_T vs WT_Oil_N, and WT_AFB_T vs WT_Oil_N (*n*=66, Bonferroni corrected) showed a stepwise model based on genotype and treatment. Note: 8 of the 9 HCC genes are in the list (underlined).

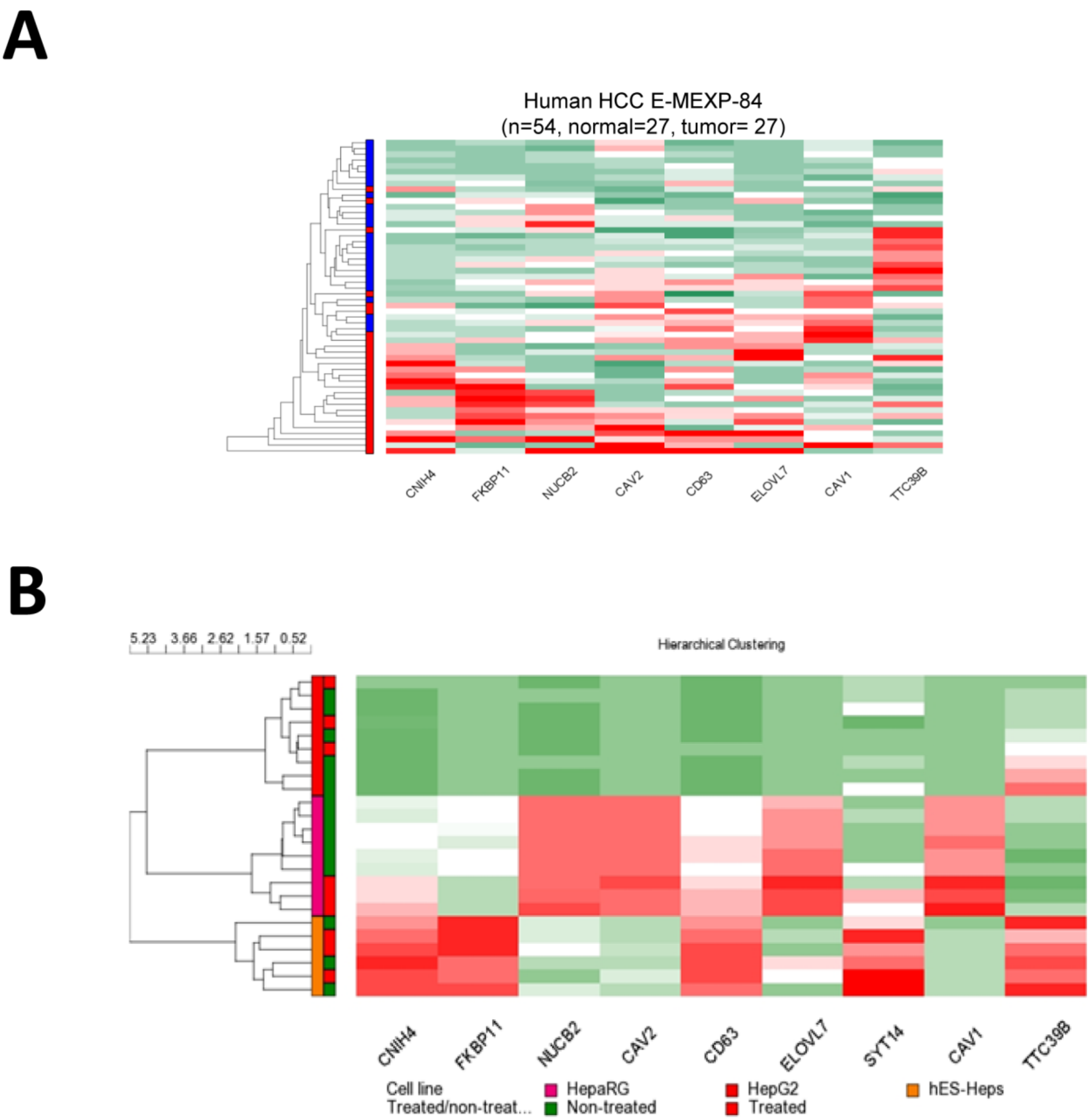

Figure S4. HCC-specific gene signature

(A,B) Differential clustering between tumor and normal HCCs was observed when segregated by the HCC gene signature pattern, similar to the mouse HCC model (A). However, the HCC gene signature was able to distinguish between primary (HepaRG or embryonic stem cell [hES]-derived) and transformed (HepG2) hepatocytes (B). ‘Treated’ refers to AFB_1 treatment.

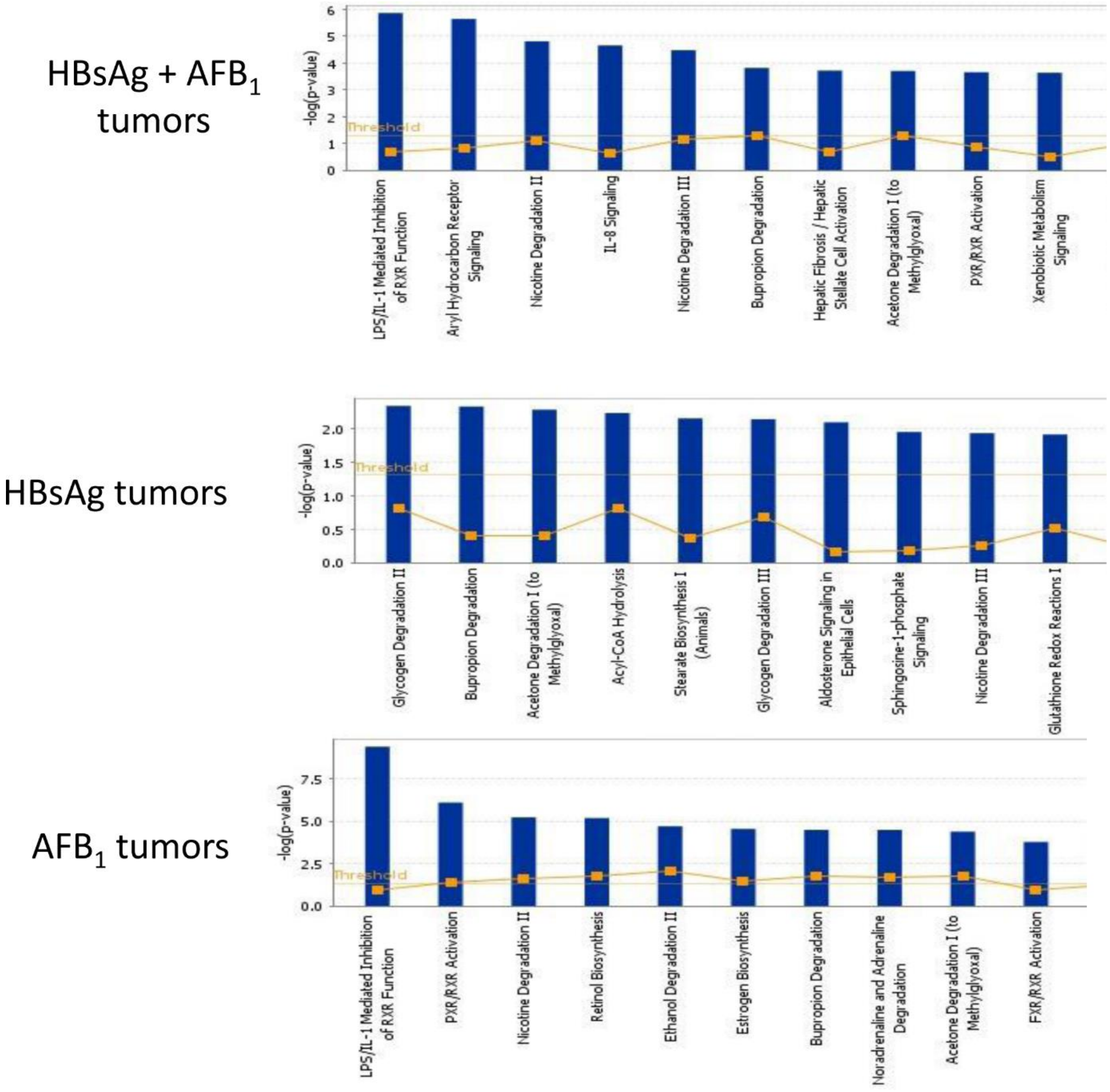

Figure S5. Molecular characterization of liver nodules from mouse models

Top canonical pathways of upstream regulators in the tumors from the various categories are indicated.

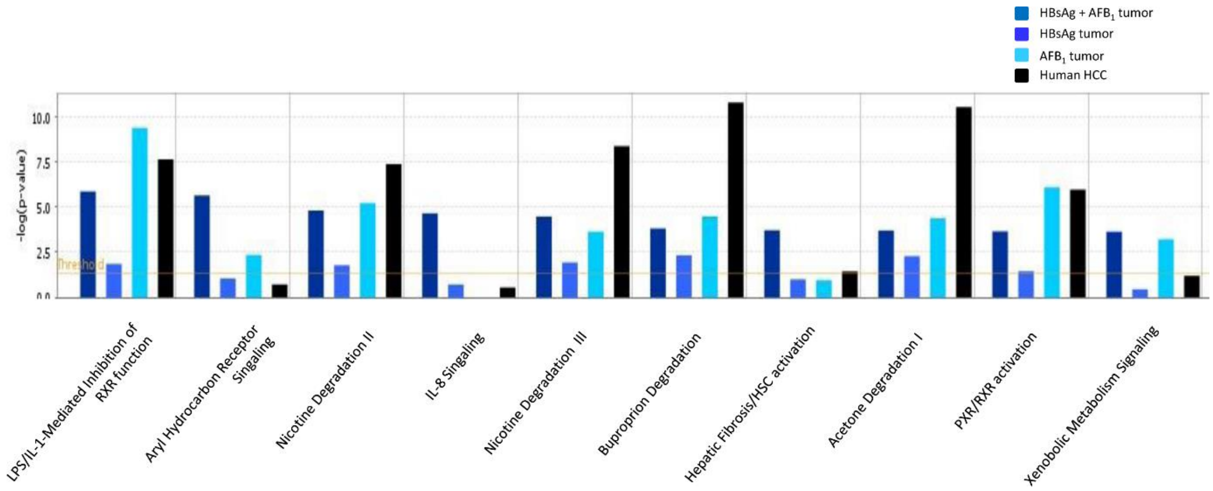

Figure S6. Molecular characterization of liver nodules from mouse models and human HCC

Direct comparison of canonical pathways affected in the human HCCs and tumors from the various mice categories are listed.

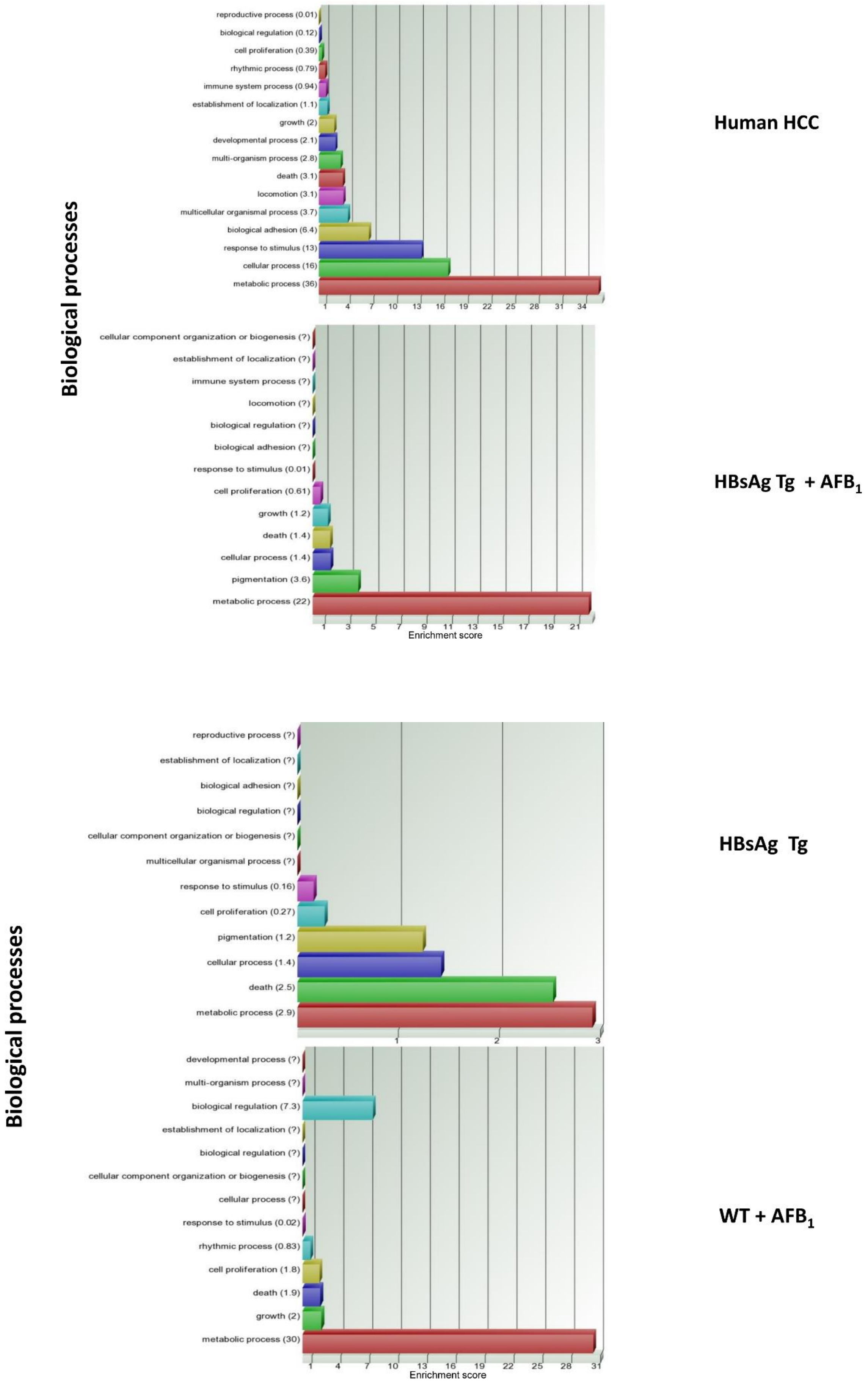
Biological processes
reproductive process (0.01)
biological regulation (0.12)
cell proliferation (0.39)
rhythmic process (0.79)
immune system process (0.94)
establishment of localization (1.1)
growth (2)
developmental process (2.1)
multi-organism process (2.8)
death (3.1)
locomotion (3.1)
multicellular organismal process (3.7)
biological adhesion (6.4)
response to stimulus (13)
cellular process (16)
metabolic process (36)
1 4 7 10 13 16 19 22 25 28 31 34
Human HCC
cellular component organization or biogenesis (?)
establishment of localization (?)
immune system process (?)
locomotion (?)
biological regulation (?)
biological adhesion (?)
response to stimulus (0.01)
cell proliferation (0.61)
growth (1.2)
death (1.4)
cellular process (1.4)
pigmentation (3.6)
metabolic process (22)
1 3 5 7 9 11 13 15 17 19 21
Enrichment score
HBsAg Tg + AFB1
Biological processes
reproductive process (?)
establishment of localization (?)
biological adhesion (?)
biological regulation (?)
cellular component organization or biogenesis (?)
multicellular organismal process (?)
response to stimulus (0.16)
cell proliferation (0.27)
pigmentation (1.2)
cellular process (1.4)
death (2.5)
metabolic process (2.9)
1 2 3
HBsAg Tg
developmental process (?)
multi-organism process (?)
biological regulation (7.3)
establishment of localization (?)
biological regulation (?)
cellular component organization or biogenesis (?)
cellular process (?)
response to stimulus (0.02)
rhythmic process (0.83)
cell proliferation (1.8)
death (1.9)
growth (2)
metabolic process (30)
1 4 7 10 13 16 19 22 25 28 31
Enrichment score
WT + AFB1

Molecular Functions

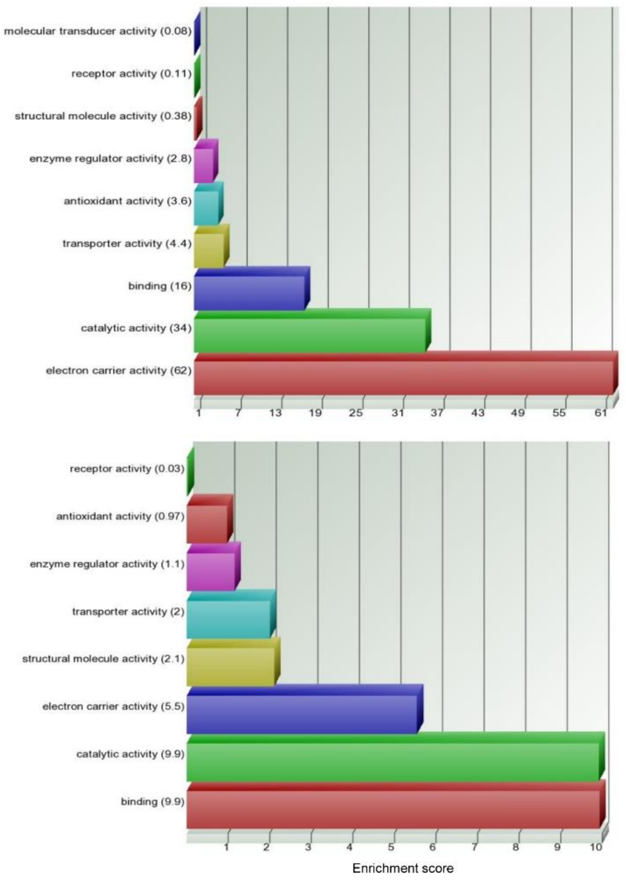

Human HCC

HBsAg Tg + AFB_1

Molecular Functions

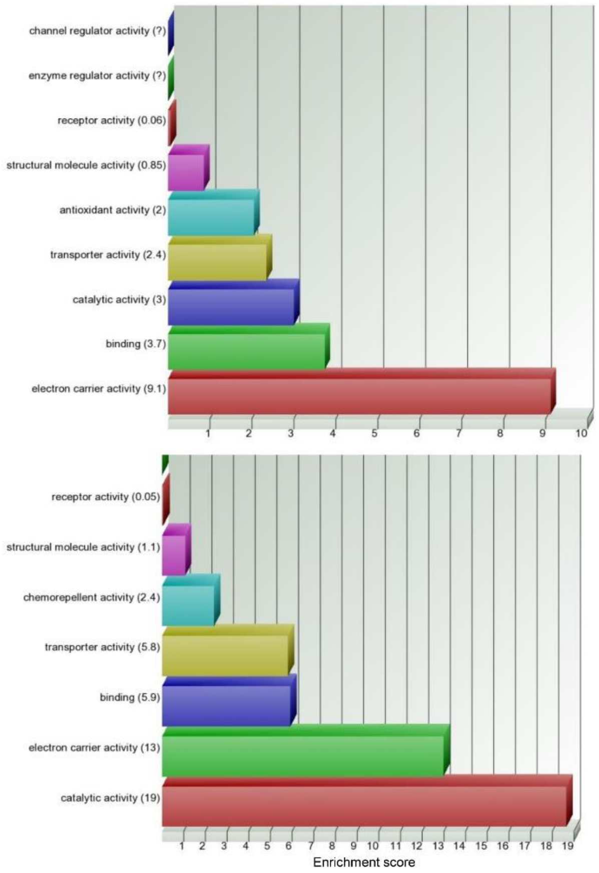

HBsAg Tg

WT + AFB_1

Cellular Components

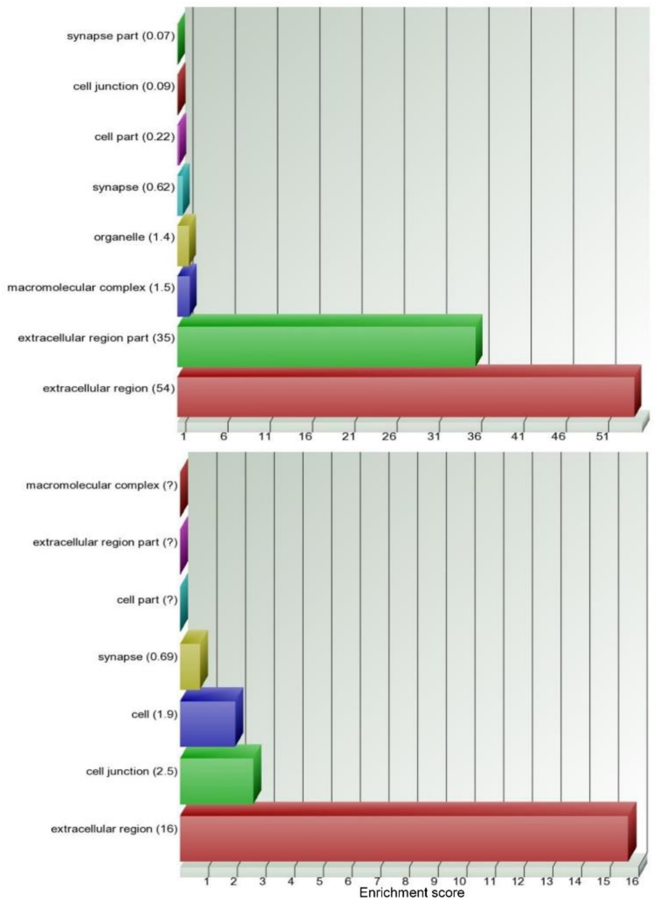

Human HCC

HBsAg Tg + AFB_1

Cellular Components

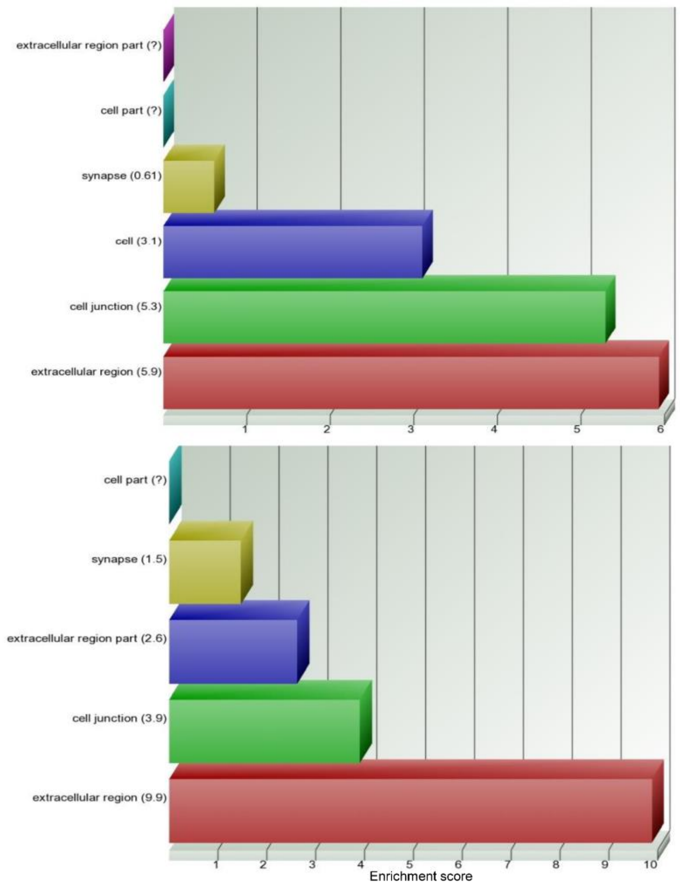

HBsAg Tg

WT + AFB_1

Figure S7. Gene Ontology analysis of molecular and cellular functions affected in liver tumors

Top biological process, molecular functions and cellular components identified through gene-ontology analysis, are shown. The major perturbations are highly similar between human HCCs and the liver nodules from the three categories of mice.

Table 1: Details of numbers of mice used in the study

A

Treatment	Genotype	Collection (months)									
		3		6		9		12		15	
		♂	♀	♂	♀	♂	♀	♂	♀	♂	♀
**Corn Oil**	**WT**	**7**	**11**	**11**	**9**	**9**	**10**	**8**	**6**	**9**	**11**
	**HBsAg Tg**	**10**	**8**	**7**	**13**	**8**	**12**	**11**	**10**	**10**	**11**
**AFB_1 at day 7**	**WT**	**9**	**8**	**9**	**9**	**8**	**10**	**8**	**9**	**8**	**12**
	**HBsAg Tg**	**8**	**7**	**15**	**10**	**11**	**15**	**13**	**14**	**13**	**15**

B

Treatment	Genotype	Collection (months)								
		9		12		15			15	
		♂	♀	♂	♀	♂	♀		♂	♀
**AFB_1 at 6 mths**	**WT**	**11**	**15**	**14**	**20**	**14**	**15**	**AFB_1 at 12 mths**	**16**	**19**
	**HBsAg Tg**	**18**	**24**	**24**	**31**	**30**	**29**		**19**	**26**

Table S1A-B - Teoh et al.

D7 injection and collection at 9 months

**Table Analyzed**	**WT ctrl vs HbsAg ctrl**			**WT ctrl vs wt AFB$_1$**			**WT ctrl vs HbsAg AFB$_1$**		
P value	1			1			0.0027		
P value summary	ns			ns			**		
One- or two-sided	Two-sided			Two-sided			Two-sided		
Statistically significant? (alpha<0.05)	No			No			**Yes**		
Data analyzed	**WT Ctrl**	**HbsAg Ctrl**	Total	**WT Ctrl**	**WT AFB$_1$**	Total	**WT Ctrl**	**HbsAg AFB$_1$**	Total
without nodules	18	17	35	18	18	36	18	14	32
with nodules	1	2	3	1	0	1	1	13	14
Total	19	19	38	19	18	37	19	27	46

**Table Analyzed**	**HbsAg Ctrl vs WT AFB$_1$**			**HbsAg Ctrl vs HbsAg AFB$_1$**			**WTAFB$_1$ vs HbsAg AFB$_1$**		
P value	0.4865			0.0104			0.0004		
P value summary	ns			*			***		
One- or two-sided	Two-sided			Two-sided			Two-sided		
Statistically significant? (alpha<0.05)	No			**Yes**			**Yes**		
Data analyzed	**HbsAg Ctrl**	**WT AFB$_1$**	Total	**HbsAg Ctrl**	**HbsAg AFB$_1$**	Total	**WT AFB**	**HbsAg AFB$_1$**	Total
without nodules	17	18	35	17	14	31	18	14	32
with nodules	2	0	2	2	13	15	0	13	13
Total	19	18	37	19	27	46	18	27	45

Table S2A - Teoh et al.

D7 injection and collection at 15 months

Table Analyzed	**WT ctrl vs HBsAg ctrl**			**WT ctrl vs wt AFB$_1$**			**WTt ctrl vs HBsAg AFB$_1$**		
P value	< 0.0001			0.0480			< 0.0001		
P value summary	***			*			***		
One- or two-sided	Two-sided			Two-sided			Two-sided		
Statistically significant? (alpha<0.05)	**Yes**			**Yes**			**Yes**		
Data analyzed	**WT Ctrl**	**HBsAg Ctrl**	**Total**	**WT Ctrl**	**WT AFB$_1$**	**Total**	**WT Ctrl**	**HBsAg AFB**	**Total**
without nodules	18	4	22	18	15	33	18	5	23
with nodules	0	17	17	0	5	5	0	23	23
Total	18	21	39	18	20	38	18	28	46

Table Analyzed	**HBsAg Ctrl vs WT AFB$_1$**			**HBsAg Ctrl vs HbsAg AFB$_1$**			**WT AFB vs HBsAg AFB$_1$**		
P value	0.0005			1			0.0001		
P value summary	***			ns			***		
One- or two-sided	Two-sided			Two-sided			Two-sided		
Statistically significant? (alpha<0.05)	**Yes**			No			**Yes**		
Data analyzed	**HBsAg Ctrl**	**WT AFB$_1$**	**Total**	**HBsAg Ctrl**	**HBsAg AFB$_1$**	**Total**	**WT AFB$_1$**	**HBsAg AFB$_1$**	**Total**
without nodules	4	15	19	4	5	9	15	5	20
with nodules	17	5	22	17	23	40	5	23	28
Total	21	20	41	21	28	49	20	28	48

Table S2B - Teoh et al.
